# Combined IMIG and Immune Ig Attenuate Allergic Responses in Beagle Dogs

**DOI:** 10.1155/2020/2061609

**Published:** 2020-04-21

**Authors:** R. M. Gorczynski, T. Maqbool, G. Hoffmann

**Affiliations:** ^1^University of Toronto, ON, Canada; ^2^Network Immunology, Vancouver, BC, Canada; ^3^Cedarlane Labs, Burlington, ON, Canada

## Abstract

**Background:**

We previously reported attenuation of serum OVA-specific IgE levels and of lymphocyte-derived IL-4, both nominal markers of allergic immunity, following injection of a combination of homologous (mouse) polyclonal anti-idiotypic immunoglobulin (Ig) and immune Ig in BALB/c mice. We predicted this might generalize to other species and using heterologous mixtures of Igs. This was assessed in mice using OVA sensitization in the presence of human Igs as a source of both anti-idiotype Ig and immune Ig and in dogs with peanut butter-induced allergic responses.

**Methods:**

Eight-week-old BALB/c mice received OVA immunization and 5 weekly injections of immune Ig or anti-idiotype Ig from either homologous (mouse) or heterologous (human) sources. Five-month-old Beagles received weekly topical exposure (on the abdomen) to peanut butter and treatment with pooled dog Ig and dog antirabies immune Ig, or a combination of human IMIG and human anti-Tet. All mice/dogs thereafter received a final allergen challenge, and serum IgG, IgE, and allergen-induced IL-2/IL-4 and IL-31 production in 72 hr cultures was measured.

**Results:**

In mice attenuation of OVA-induced allergy (IgE-specific Ig and OVA-induced IL-4) was seen using both mouse and human Ig mixtures, without effect on OVA serum IgG or OVA-induced IL-2. Attenuation of concanavalin A- (ConA-) induced IL-4 : IL-2 production and of peanut butter-induced IL-4 and IL-31 was seen in dogs receiving combinations of both heterologous and homologous immune Igs and anti-idiotype Igs, with no decline in IL-2 production. Allergen-specific IgE/IgG was not detectable in dog serum, but there was a trend to lower total serum IgE levels (and decreased IgE : IgG ratios).

**Conclusion:**

Homologous and heterologous combinations of polyclonal IMIG and immune Ig attenuate allergic responses in mice and dogs. This treatment protocol represents a novel approach which can be adapted for allergic desensitization in veterinary and human use.

## 1. Introduction

Allergic rhinitis, asthma, and atopic eczema are among the commonest causes of chronic ill health, with a combined prevalence of 10-20% [1–3]. All such diseases are increasing in prevalence, with considerable burden to total health care costs. While the tendency to develop allergic reactions is lifelong, the actual manifestations of disease change over time. As an example, children often develop eczema early, followed by allergic rhinitis and asthma. All allergic diseases impact quality of life and, in more extreme cases, morbidity and even mortality.

Currently, treatment for these diseases follows several major guiding principles [1], including allergen avoidance, drug therapy (including oral and topical potent anti-inflammatory medications (steroids), antihistamines, and in severe cases with anaphylaxis, adrenalin), and finally, where the allergen is identifiable, desensitization therapy [4]. The latter uses controlled exposure to escalating doses of purified allergen to alter the body's reaction to allergen exposure. In general, desensitization therapy treatment is given long term and often still in association with conventional allergy-relieving medications. There remains an unmet need to develop novel therapies.

Given concerns regarding extrapolation of allergy studies from mouse to man [5], there has been a growing interest in the analysis of allergic reactivity in other animal species. Humans and their most important domestic animals, namely, cats and dogs, have a similar IgE receptor repertoire and expression pattern, with essentially similar cell types known to be implicated in the triggering and/or regulation of allergic responses, including mast cells, eosinophils, and regulatory T cells. Such animals have thus been favoured for preclinical research studies.

Skin, respiratory, and food allergies are not uncommon in dogs, and the immune mechanisms involved are more like humans than in rodents [5, 6], making them a favoured species for study [7–11]. Dust mite allergens are thought to be highly relevant to canine allergic responses [12], although skin reactivity and dust mite-specific serum IgE have also been detected in several dogs without clinical signs of allergy, suggesting that sensitization can occur without necessarily inducing clinically significant reactivity [13]. Dogs develop atopic dermatitis as a result of sensitization to storage mites [14] and plant-derived allergens [15], as well as to flea allergens [16] —though less so to other insects—and even moulds [17]. In all cases, IgE responses to several identified protein allergens have been documented. Induction of IL-31 is a prominent factor in the development of atopic dermatitis in dogs [18].

We reported on the use of a combined injection of polyclonal anti-idiotype antibodies, along with polyclonal immune antibodies, on resetting immune regulatory networks in rodents [19]. This treatment regulated many immune reactivities, with decreased inflammatory cytokines in inflammatory colitis, decreased skin graft rejection in a transplant model, and decreased IgE and IL-4 sensitization in a rodent allergy model (to ovalbumin (OVA)). The antigen-specific regulation seen was independent of the specific antigens used to prepare the polyclonal immune Ig. One mechanism responsible for these effects involved perturbation of regulatory T cell networks, consistent with other data favouring a role for Tregs in control of allergic reactivity [20]. We have used the Beagle model described earlier [7] to assess whether a mixture of homologous antibodies (pooled dog Ig as a source of anti-idiotype; pooled dog rabies immune globulin as a source of immune Ig) would attenuate allergic responses in dogs. In this model, application of peanut butter paste caused enhanced serum IgE and when animals were then challenged orally, pruritic dermatitis, eosinophilic dermatitis, and IgE-positive cells in skin were seen in atopic dogs [7]. We asked whether the treatment protocol used to attenuate allergic responses in mice decreased allergen-induced IL-4 and IL-31 production in sensitized dogs, without diminishing an IL-2 (nonatopic) response in the same animals. We also explored whether heterologous serum Ig mixtures (pooled polyclonal human Ig and pooled polyclonal human anti-tetanus Ig) would lead to similar allergic desensitization.

## 2. Materials and Methods

### 2.1. Preliminary Studies in Mice

#### 2.1.1. Mice and Ethics Review

All mice were bred at Cedarlane labs from Jax founder stock. All studies were approved by a local institutional review board, certified by the Canadian Council on Animal Care. Animals were maintained under SPF conditions throughout the study.

Immunization of BALB/c mice to produce IgE against OVA was described elsewhere [19]. Eight mice per group received 10 *μ*g OVA emulsified in alum at day 0 and day 10. One control group received no OVA immunization. All but one OVA-immunized group and the no OVA control received ongoing exposure to egg white solution in the drinking water for 10 d until sacrifice—the other control group received OVA immunization only (no EWS). Beginning on day 7, groups of mice given OVA and EWS received 5 weekly 10 *μ*g intramuscular (IM) injections of pooled polyclonal BL/6 anti-C3H immune Ig, polyclonal C3H anti-anti-C3H Ig (C3H anti-BL/6 absorbed with BL/6: anti-idiotype Ig), or a combination of these latter 2 Ig preparations (estimated ~0.5 mg/kg/dose). At 42 d, all mice received a booster injection of OVA in alum, with sacrifice 7 d later. In a separate study, mice received OVA stimulation in the same fashion, but the Ig preparations used for treatment were heterologous in nature (pooled human IVIG, given intramuscularly, hence IMIG) and pooled human anti-Tet immune Ig (both purchased from Grifols, USA). In trial studies, we have found that optimal dosing for this heterologous Ig used as anti-idiotype (IMIG) was ~3x higher than the purified mouse polyclonal C3H anti-anti-C3H Ig, and accordingly, IMIG was used at a dose of ~1.5 mg/kg/dose. It should be noted that in separate studies (not shown), we have assessed different routes of delivery of immunomodulatory Igs, including intravenous (iv), intraperitoneal (ip), and subcutaneous (sq), without significant variation in efficacy, although anti-idiotype and immune Igs must be given by separate routes for optimal effects.

Serum was obtained from mice at sacrifice by cardiac puncture [19]. At the same time, single-cell splenocyte preparations from individual animals were resuspended after washing (800g × 5 min at 4°C) in RPMI with 10% fetal calf serum (RPMI_10_). 5 × 10^6^ splenocytes from individual animals were incubated in duplicate in vitro in 2 ml RPMI_10_ with 0.1 mg/ml OVA for 72 hr, and culture supernatants assayed by ELISA for IL-2 and IL-4 production.

OVA-specific IgE or IgG was measured in all serum samples by ELISA using plates coated with 100 ng/well of OVA and developed with HRP-anti-mouse IgE or HRP anti-IgG, followed by appropriate substrate.

### 2.2. Dogs

Beagle dogs, aged 5 months at initiation of use, were purchased by CARE research (Fort Collins, Colorado) and housed in their facility, under supervision by approved veterinary services, and in accordance with a registered animal protocol committee. Animals were fed approved dog chow ad libitum, with daily exercise. Weights were followed 3x/week, with veterinary inspections weekly. Dogs were released for companion pets at the termination of the study. There were no significant differences in weights pre-/posttreatment for any of the groups of dogs used in the study.

#### 2.2.1. Ig Preparations for Injection

Pooled human IVIG and anti-Tet Ig were purchased from Grifols, USA. Pooled Beagle serum was purchased from BioIVT (Westbury, NY), and Ig was isolated following ammonium sulphate precipitation. Polyclonal dog rabies immune Ig was pooled from the serum (3 ml/animal) obtained, with the owner's consent, from 12 outbred dogs, 8-24 months of age, boosted with rabies vaccine 4 weeks before.

Groups of 5 Beagle dogs received IM gluteal injections in 0.5 ml sterile PBS of 12 mg/dose of pooled polyclonal Ig (dog IMIG-group 2, or human IMIG-group 3) and 4 mg IM in the opposite limb of 0.5 ml sterile PBS of polyclonal immune Ig (dog antirabies-group 2 or human anti-Tet-group 3). Control dogs (group 1) received only dog IMIG (total Ig used 16 mg/dose). Animals received weekly injections ×5 weeks, followed by 3 further injections at 2-week intervals. Beginning on the first day of injection, all animals received ~15 gm peanut butter paste weekly smeared on their abdomens. A collar was applied for ~6-8 hours after peanut butter application to prevent dogs from removing the peanut butter. Five days following the last IM injection, all animals received an oral challenge with peanut butter (~15 gm). Photographic images of the abdomen were taken daily for 3 days after oral challenge. Samples of heparinized blood (4 ml/dog) were harvested at the outset of the study, 3 days after oral challenge, and flown (FEDEX) at room temperature to Cedarlane Labs, Ontario, Canada, for analysis. All samples were received and harvested for serum/PBMCs for culture, within 24 hr of collection. All individuals at CARE were blinded to which treatment contained which individual dogs. During the study, 3 dogs (1 in each group) developed, at different times in the study, a minor skin irritation at the site of injection. In each case, the dogs were monitored by a CARE veterinarian, and the irritation resolved without treatment within 7-10 days, with no recurrence.

### 2.3. Dog Serum IgG and IgE Assays and IL-4, IL-2, and IL-31 Cytokine Assays

PBL harvested in heparinized tubes was diluted 1 : 1 with PBS, layered over 4 ml Ficoll/Hypaque, and centrifuged for 20 min at 1600 rpm at room temperature (rt). The diluted serum (1 : 1) overlaying the cell interface was collected with a Pasteur pipette, and the cells (PBMCs) were diluted into 10 ml RPMI with 5% fetal calf serum+5% dog serum (both sera heat inactivated 30 min at 56°C: hereafter RPMI_sera_). This sample was centrifuged at 1600 rpm for 10 min at rt. The cell pellet was resuspended in 2 ml RPMI_sera_, and cells were diluted in RPMI_sera_ to a concentration of 2.5 × 10^6^/ml. Serum samples were stored at -80°C for later Ig analysis (ELISA). 1.5 × 10^6^ PBMCs were stimulated in duplicate in 1.5 ml RPMI_sera_ in 24-well culture plates with either 5 *μ*g/ml ConA (time zero and end of study) or with 20 *μ*l of DMSO extract of peanut butter (1 gm in 10 ml DMSO: final concentration in culture ~2 mg/ml); control samples were incubated with 1.5 ml RPMI_sera_ and 20 *μ*l DMS only. Supernatants were harvested from culture at 72 hr for IL-2/IL-4 detection by ELISA.

IgG and IgE concentrations in all sera were determined by ELISA as follows. Goat anti-dog IgG was purchased from Thermo Scientific, Fisher, Canada (Cat # 18763). Goat anti-dog IgE was from Bio-Rad, Canada (Cat # AHP946). Donkey anti-goat HRP was obtained from R&D Systems Canada. High-binding ELISA plates were from SARSTEDT (Cat # 82.1581.200) and ELISA substrate solution from Thermo Scientific (Cat #34028). 100 *μ*l aliquots of the experimental sera were diluted 1 : 100, 1 : 1000, and 1 : 10,000 and added in quadruplicate to microtiter plates in 100 *μ*l. All microtiter plates were coated overnight (37°C), along with duplicate control wells containing known concentrations of standard amounts of dog IgG or dog IgE (both purchased from R&D Systems). Thereafter, a routine ELISA was performed using steps including addition of anti-dog IgG or anti-dog IgE, followed by donkey anti-goat HRP and later addition of a substrate, with ultimate reading in an ELISA plate reader. IgG and IgE levels in test sera were determined from a standard curve using purified IgG and IgE.

IL-2 and IL-4 levels in ConA-activated or peanut butter-stimulated PBMCs were determined using commercial kits provided by R&D (Duo Set IL-2: Cat # 1815; Duo Set IL-4: Cat # DY754), along with auxiliary reagent kits (R&D Cat # DY 008). IL-31 was similarly assayed using a commercial kit with reagents/standards, from NeoScientific (Cat #C10041).

### 2.4. Statistics

All data from mouse experiments reported below are summed over at least two studies, with a minimum of 16 mice in all groups for all experiments. In general, for studies with multiple groups, a multivariate analysis of variance (MANOVA) test was first applied to assess for any significant differences between groups, and subsequently, where indicated, paired *t*-tests were used to compare individual groups with the documented control.

Dog studies were performed using the 3 groups described. Five animals/group were randomly assigned to the different treatments. Comparisons between groups were by MANOVA.

## 3. Results

### 3.1. OVA-Specific IgE, but Not IgG, Is Attenuated in OVA-Primed Mice by Combined Treatment with Pooled Anti-idiotype and Immune Ig, Even with a Heterologous Source of Igs

**Figure 1:**
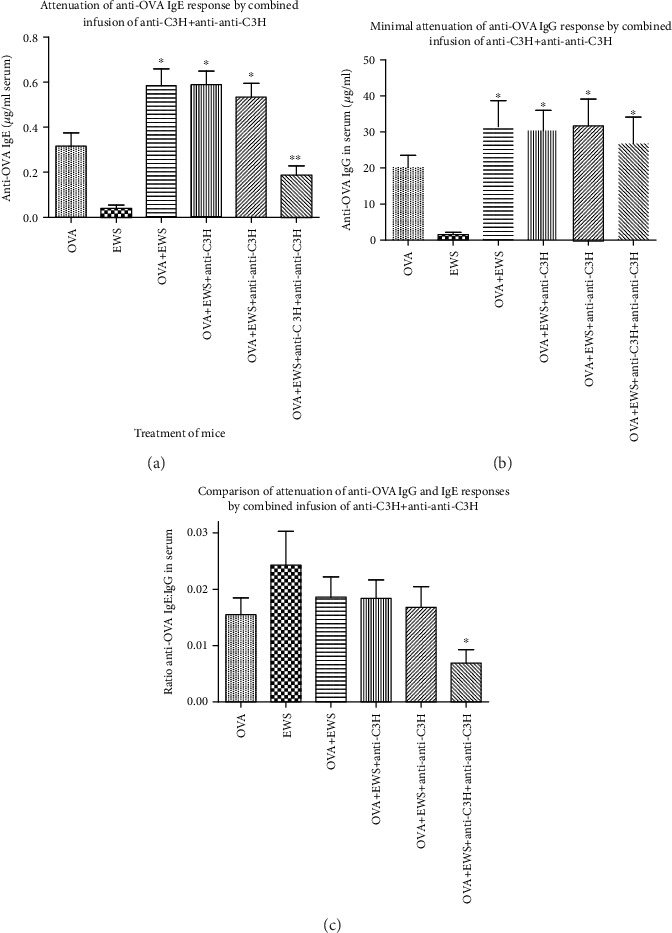
OVA-specific lgE ((a) mean ± SD) and lgG (b) in mice immunized with OVA and immune or anti-idiotype sera (or both). OVA-immunized mice except for the control group (far left) drank EWS. (c) The ratio of ^∗^*p* < 0.05 compared with OVA only; ^∗∗^*p* < 0.05 compared with OVA+EWS.

**Figure 2:**
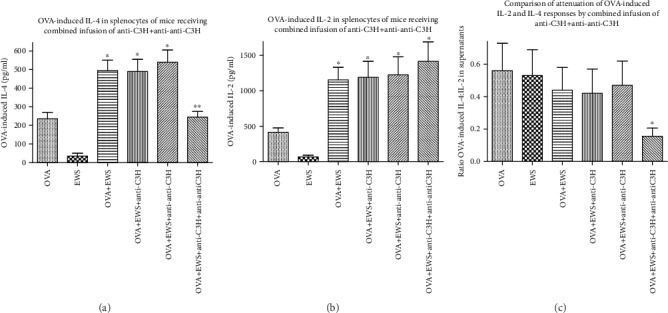
OVA-induced IL-4 production ((a) mean ± SD) or IL-2 production (b) at 72 hr in OVA-stimulated splenocytes from mice of Figure 1. Data in (c) show changes in the induced IL-4 : IL-2 ratios in supernatants harvested from OVA-stimulated cells of the different groups. ^∗^*p* < 0.05 compared with OVA only; ^∗∗^*p* < 0.05 compared with OVA+EWS.

Our first studies repeated the findings previously reported [19]. BALB/c mice were immunized with OVA in alum and given EWS in the drinking supply as a source of persistent allergen exposure. Groups of mice also received 5 weekly IM injections of anti-idiotype and/or immune Igs as indicated in Materials and Methods. Following a final boost with OVA after these 5 Ig injections, mice were sacrificed 7 d after OVA, and serum IgG/IgE to OVA was detected by ELISA, with IL-2 and IL-4 levels detected at 72 hr from OVA-stimulated splenocytes. Data pooled from two such studies, using 8 mice/group, are shown in Figures 1 and 2.

Comparison of Figures 1(a) and 1(b) shows clearly that mice exposed to EWS following OVA immunization developed a greater IgG and IgE response than mice given OVA immunization only. Mice exposed to EWS alone (no OVA immunization) produced little OVA-specific Ig. Treatment of mice with the combined Ig preparations, but not either Ig preparation alone, led to the attenuation of the IgE response (Figure 1(a)), but not the IgG response (Figure 1(b)). The effect produced by the combined Ig preparations is emphasized by comparison of the OVA-specific IgE : IgG ratios in all groups (Figure 1(c))—attenuation occurred only in the group receiving combined Ig treatment.

OVA-induced IL-4 and IL-2 production looked like the IgE/IgG levels in all groups. Treatment with the combined Ig preparations led to the attenuation of IL-4 levels after OVA stimulation (Figure 2(a)), but not IL-2 levels (Figure 2(b)), an effect emphasized by assessment of IL-4 : IL-2 ratios in these groups (Figure 2(c)).

**Figure 3:**
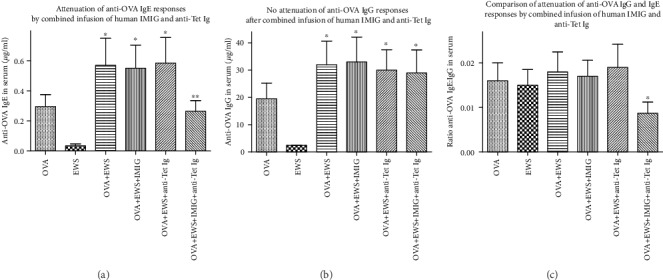
As for Figure 1, except that in this case all mice received heterologous Ig treatments (human-derived IMIG and/or pooled human immune anti-Tet Ig) not homologous immune or anti-idiotype Igs. ^∗^*p* < 0.05 compared with OVA only mice; ^∗∗^*p* < 0.05 compared with the OVA+EWS group.

**Figure 4:**
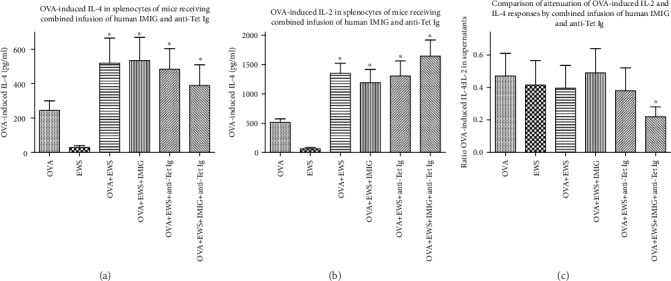
Attenuation of OVA-induced IL-4 production (a) but not IL-2 production (b) at 72 hr in OVA-stimulated splenocytes from mice in Figure 3. Data in (c) show changes in the calculated induced IL-4 : IL-2 ratios in supernatants harvested from OVA-stimulated cells of the different groups. All data show mean ± SD of triplicate cultures using splenocytes harvested from 8 mice/group.

We had predicted that in vertebrates with a shared ancestral immune system, even heterologous Igs (immune+anti-idiotype) might produce the same effects as mixtures of homologous Igs [19, 21, 22]. Accordingly, OVA-immunized mice were treated with polyclonal immune human Ig (anti-Tet Ig) and/or polyclonal human anti-idiotype Ig (using IVIG as a source of this [22, 23]—since delivery was intramuscular, this is henceforth referred to as IMIG). Data in Figures 3 and 4 are pooled from 2 such studies.

Comparison of Figures 1–4 shows that the effects seen using homologous (mouse) combinations of immune Ig and anti-idiotype Ig were recapitulated using heterologous (human) reagents as the antibody source.

### 3.2. Effect of Combinations of Immune Ig and Anti-idiotype Ig on Peanut Butter-Induced Sensitization in Beagle Dogs

**Figure 5:**
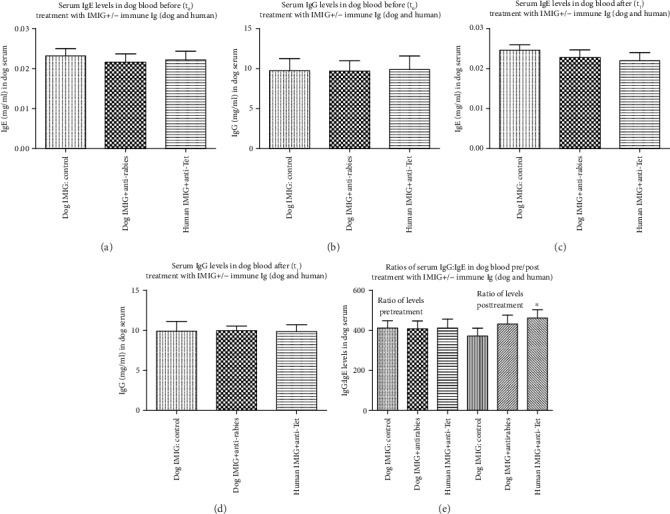
Serum IgE and IgG (mean ± SD) in dogs exposed to be peanut butter and treated with dog IMIG/dog immune Ig, or human IMIG/human immune Ig. Controls received only dog IMIG injection. Levels are shown at pretreatment (a, b) and posttreatment (c, d). (e) The ratio of IgG : IgE in all groups. ^∗^*p* < 0.05 compared with posttreatment IMIG controls.

**Figure 6:**
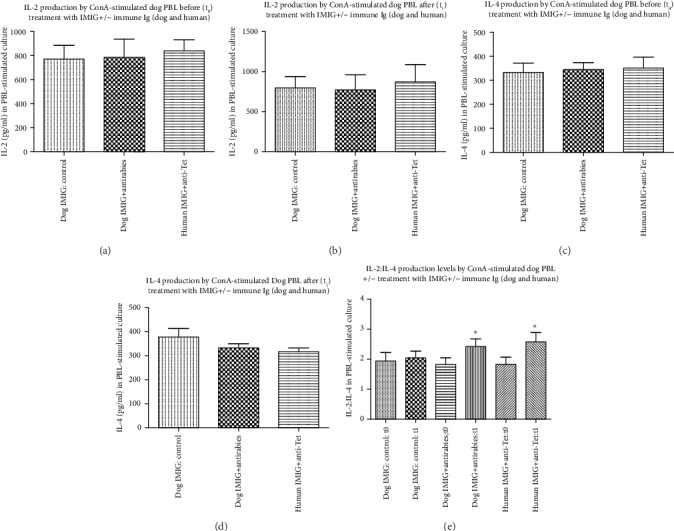
Levels of ConA-induced IL-2 and IL-4 (mean ± SD) in 72 hr cultures of PBMCs from groups dogs from Figure 5. PBMCs were cultured at pretreatment (a, c) and posttreatment (b, d). (e) The ratio of IL-2 : IL-4 in all groups. ^∗^*p* < 0.05 compared with equivalent IMIG controls.

Groups of 5/group 8 kg Beagle dogs receiving topical exposure to peanut butter were given 5 weekly injections of dog immunoglobulins (pooled IMIG and antirabies immune Ig) or human immunoglobulins (pooled IMIG or anti-Tet immune Ig) as described in Materials and Methods. After one final oral challenge with peanut butter, serum and PBMCs were harvested and total dog serum IgE and IgG levels were determined by ELISA (Figure 5)—for technical reasons, we were unable to detect the presence of peanut butter specific dog IgE or IgG. In addition, we measured ConA-induced IL-2 and IL-4 levels assayed from PBMC cultures of both pre- and posttreatment dog samples stimulated (see Materials and Methods) with 5 *μ*g/ml ConA (Figure 6). Peanut butter-induced IL-2, IL-4, and IL-31 levels were measurable only on posttreatment dog samples, using PBMCs stimulated with 2 mg/ml (estimated) DMSO peanut butter extract.

**Figure 7:**
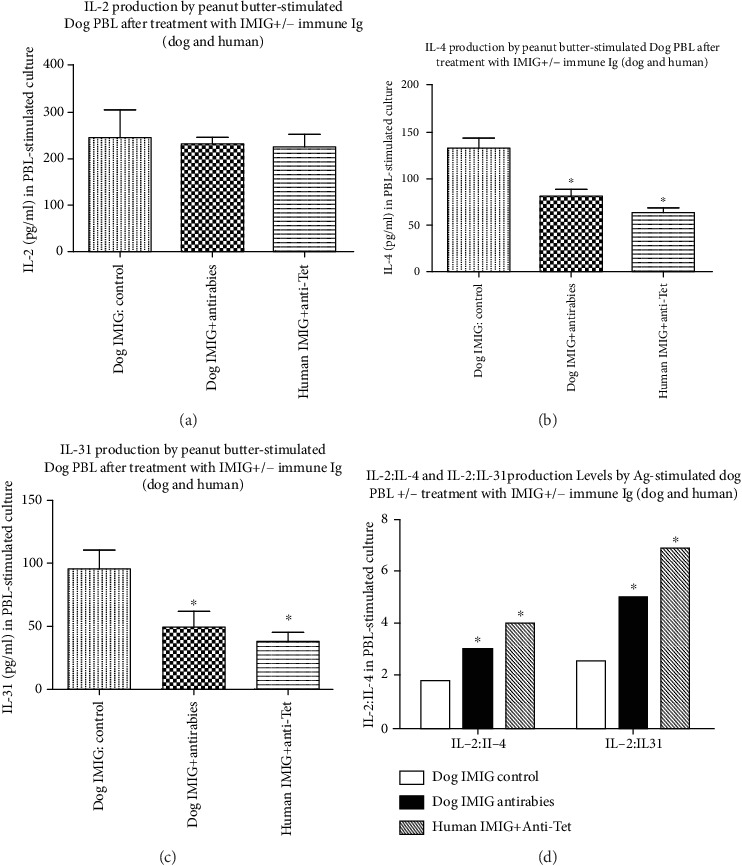
Peanut butter-induced IL-2, IL-4, and IL-31 (a–c) in stimulated PBMCs from Figures 5 and 6. No IL-2/IL-4/IL-31 production was detected from stimulated PBMCs from dogs pretreated with peanut butter or from posttreatment PBMCs stimulated with DMSO vehicle only (not shown). (d) The ratio of IL-2 : IL-4 and IL-2 : IL-31, respectively, in all groups. ^∗^*p* < 0.05 compared with equivalent IMIG controls.

In Figure 7, no IL-2/IL-4/IL-31 production was seen in these samples after culture with DMSO vehicle alone.

Firstly, there were no significant differences in overall IgE or IgG levels in any groups before/after treatment (Figures 5(a)–5(d)). There was a trend to an increased IgG to IgE ratio after peanut butter sensitization in dogs treated with the combined dog or human Igs, which reached significance for the group treated with human Igs (Figure 5(e)), implying a relative decrease in IgE levels (to IgG) in this group. We suspect purely for technical reasons we were unable to detect any dog-specific IgE or IgG levels in any groups after sensitization. A generalized decrease in allergic reactivity in the groups treated with combined Ig infusions was suggested by analysis of IL-2 and IL-4 release in response to ConA in various groups. While there was no significant difference in IL-2 production in the various groups before or after treatment, with a generalized trend to increased IL-2 production in all groups after prolonged peanut butter exposure (Figure 6(b) vs.  Figure 6(a)), there was a trend to decreased IL-4 production after treatment with combined dog or human Igs (Figure 6(d)), which again reached significance for the group treated with human Ig. Comparison of IL-2 : IL-4 ratios in all groups before/after treatment (Figure 6(e)) confirmed a relative decrease in IL-4 production in sensitized groups treated with either dog or human Igs. Finally, analysis of allergen-induced IL-2, IL-4, and IL-31 production in sensitized dogs showed significant differences only in IL-4/IL-31 production (not IL-2 levels) in dogs treated with dog or human Igs (Figure 7(a)), which again translated to a highly significant increase in IL-2 : IL-4 and IL-2 : IL-31 ratios in the same groups (Figure 7(b)).

## 4. Discussion

Exploration of novel therapies for treatment of allergic diseases has highlighted measures which decrease IgE levels and target cytokines (e.g., IL-4, IL-5, and IL-31) thought to be implicated in control of those levels and other cell populations (eosinophils/basophils) implicated in allergic disease [18, 24–30]. We reported on the use of a novel therapy aimed at manipulating self-reactive immune regulatory networks through deliberate perturbation of idiotype : anti-idiotype interactions, as a potential mechanism to attenuate several pathological immune reactions in rodents, including allergic sensitization to ovalbumin [19]. To date, no investigations of the effects of these treatments in larger animals or humans have been performed.

We hypothesized that attenuation of mouse allergic responses to ovalbumin following repeated injection with immune Ig and anti-anti-self Ig (Figures 1 and 2) was best understood in terms of an effect mediated by a resetting of both B cell and T regulatory (Treg) cell immune networks in mice receiving combined immune Ig and anti-idiotype Ig [19, 22]. We have shown furthermore that in rodents, the mechanism(s) implicated in the attenuation of allergic responses after such treatment critically involves augmentation of Treg activity induced by combinations of immune Ig and anti-idiotype Ig [22, 31]. We had predicted that in vertebrates with a shared ancestral immune system, even heterologous Igs (immune+anti-idiotype) would produce the same effects [21, 22]. Testing this hypothesis initially in a mouse model using BALB/c mice sensitized with ovalbumin showed that attenuation of allergic responses was seen in mice receiving commercial immune Ig and anti-idiotype Ig of human origin, see Figures 3 and 4. These important observations justified a pilot preclinical trial of this same therapy in a dog model (Beagles) of atopic dermatitis, the most common allergic skin disease in dogs [5–10].

Dogs sensitized to peanut butter [7] showed evidence for a generalized relative decrease in serum IgE : IgG levels following combined treatment with the two Ig preparations, along with a similar attenuation of the IL-4 : IL-2 ratio from ConA-stimulated PBMCs (Figures 5 and 6). More dramatically, allergen-induced IL-4 and IL-31 production and IL-4 : IL-2 and IL-31 : IL-2 levels were decreased in treated animals (Figure 7). The attenuation of IL-31 production following this treatment is particularly interesting given the evidence that IL-31 is a key cytokine implicated in itching in allergic dogs and that mAb directed to IL-31 has been licenced for use in the treatment of allergic dermatitis [18], while vaccination against IL-31 has also been proposed as a novel therapy in such animals [30]. Somewhat surprisingly, the effects we saw on the attenuation of IL-4/IL-31 production were most pronounced using combinations of human immune Ig and anti-idiotype Ig (commercial anti-Tet and IMIG) rather than combinations of dog reagents (antirabies IgG and pooled IMIG). We suspect this may simply reflect an unexplored dose-response effect, which is already under investigation. It is important to note that we were unable to detect any allergen-specific IgE or IgG in peanut butter-sensitized dogs, although this failure is hypothesized to be more technical than real, particularly given evidence for allergen-induced IL-2 and IL-4 in the same animals (Figure 7). We are in the process of assessing responses in dogs treated with a purified allergen (dust mite) to clarify this issue further. Our data are consistent with the notion that the attenuation of allergen-induced IL-4/IL-31 responses by the therapy used is instrumental in the effects seen, as suggested elsewhere [18, 19, 26, 28, 30].

In summary, we have extended our previous studies which reported on a novel therapy which could attenuate allergic immunity in rodents, to show that the same approach is efficacious in suppressing such immunity in large animals (dogs), a result which has important implications for the veterinary community.

## Data Availability

Data and materials (where available) included in this study will be made freely available.
